# The Radiological Mimicry of Ring-Enhancing Lesions: Cerebral Toxoplasmosis Masquerading as Metastatic Disease

**DOI:** 10.7759/cureus.92604

**Published:** 2025-09-18

**Authors:** Sai S Bhiraju, Emil John, Raamish Asad Raza, Mashrur Ahmed, Lior Fengas, Sharafath Hussain Zahir Hussain

**Affiliations:** 1 Medicine, Milton Keynes University Hospital, Milton Keynes, GBR; 2 Emergency Medicine, Poole General Hospital, University Hospitals Dorset NHS Foundation Trust, Poole, GBR; 3 Acute Medicine, Milton Keynes University Hospital, Milton Keynes, GBR

**Keywords:** cerebral toxoplasmosis, cns lymphoma, hiv/aids, hiv-associated neurocognitive disorders, hiv neuroinfections, neuroradiology, ring-enhancing lesions

## Abstract

A 42-year-old man presented with progressive confusion and was found to have multiple intracranial lesions with surrounding vasogenic oedema on non-contrast computed tomography (CT), initially raising suspicion for metastatic brain disease. Subsequent contrast-enhanced magnetic resonance imaging (MRI) revealed multiple ring-enhancing lesions, indicating the consideration of alternative diagnoses, particularly opportunistic infections. Further workup confirmed undiagnosed HIV/AIDS with severe immunosuppression and positive *Toxoplasma gondii* serology. A diagnosis of cerebral toxoplasmosis was made. The patient demonstrated significant clinical and radiological improvement following appropriate antiparasitic therapy. This case illustrates the pivotal role of radiologist's insights with neuroimaging in guiding differential diagnosis. It enabled early distinction between neoplastic and infectious etiologies and ultimately directed life-saving treatment in patients with atypical intracranial lesions.

## Introduction

Intracranial ring-enhancing lesions often prompt a broad differential diagnosis that includes neoplastic causes (such as primary brain tumours or metastases) and non-neoplastic causes (including abscesses, granulomas, or demyelination) [1]. In immunocompetent adults, multiple ring-enhancing brain lesions are most commonly associated with metastatic cancer [1]. In contrast, in patients with HIV/AIDS, opportunistic infections such as cerebral toxoplasmosis and primary central nervous system (CNS) lymphoma are the predominant causes [2]. *Toxoplasma gondii* encephalitis, also referred to as cerebral toxoplasmosis, is the most common CNS infection in AIDS patients who are not on prophylaxis [3]. These patients typically present acutely with lethargy, headache, confusion, seizures, or focal neurologic deficits [3]. Distinguishing infectious lesions from malignancy on imaging is crucial, as misclassifying an abscess for a tumour can delay life-saving therapy [4]. Prompt treatment of cerebral toxoplasmosis can lead to dramatic clinical improvement; over 70% of patients show a significant response within two weeks of appropriate therapy [4]. This case exemplifies such a diagnostic dilemma, in which initially suspected brain metastases were ultimately identified as cerebral toxoplasmosis. It adds to the growing literature on CNS "tumour mimics", emphasising the need for radiologists to consider opportunistic infections in the differential diagnosis of intracranial lesions [5]. 

## Case presentation

A 42-year-old man, a recent migrant from Ghana, with unknown past medical history, was brought to the emergency department by a friend due to a two-week history of confusion and mutism. According to collateral history, he had been progressively withdrawn, minimally verbal, and increasingly disoriented for the last three weeks. There was no documented history of malignancy or chronic illness. 

On examination, the patient was afebrile but significantly confused and non-verbal. He was disoriented to time, place, and person. Neurological examination revealed blunted affect, impaired attention, and global cognitive dysfunction. No focal neurological deficits were appreciated. Cranial nerve examination was grossly intact. There was no meningism, and motor and sensory examination were unremarkable. Fundoscopy showed no papilloedema. Systemic examination was otherwise within normal limits. Admission bloods did not show any major abnormalities (Table 1).

**Table 1:** Lab values EGFR: estimated glomerular filtration rate

Parameters	Reference range	Value on admission	Value on discharge
Haemoglobin (g/L)	130-170	148	135
White blood cell (×10^9^/L)	3.7-11	3.6	9.6
Platelet (×10^9^/L)	150-400	463	323
Neutrophil (×10^9^/L)	1.7-7.5	1.8	8.2
Lymphocyte (×10^9^/L)	0.9-4	1.1	0.7
Monocyte (×10^9^/L)	0.2-1	0.6	0.8
Eosinophil (×10^9^/L)	0-0.5	0.1	0
Basophil (×10^9^/L)	0-0.1	0	0
Sodium (mmol/L)	133-146	135	134
Potassium (mmol/L)	3.5-5.3	3.9	4.4
Urea (mmol/L)	2.5-7.8	7.1	6.6
EGFR (mL/min/1.73 m²)	>90	69	78
Alanine aminotransferase level (IU/L)	0-50	12	39
Gamma-glutamyl transpeptidase (IU/L)	0-54	53	77
Alkaline phosphatase (IU/L)	30-130	56	52
C-reactive protein (mg/L)	0-5	25	0.4

**Figure 1:**
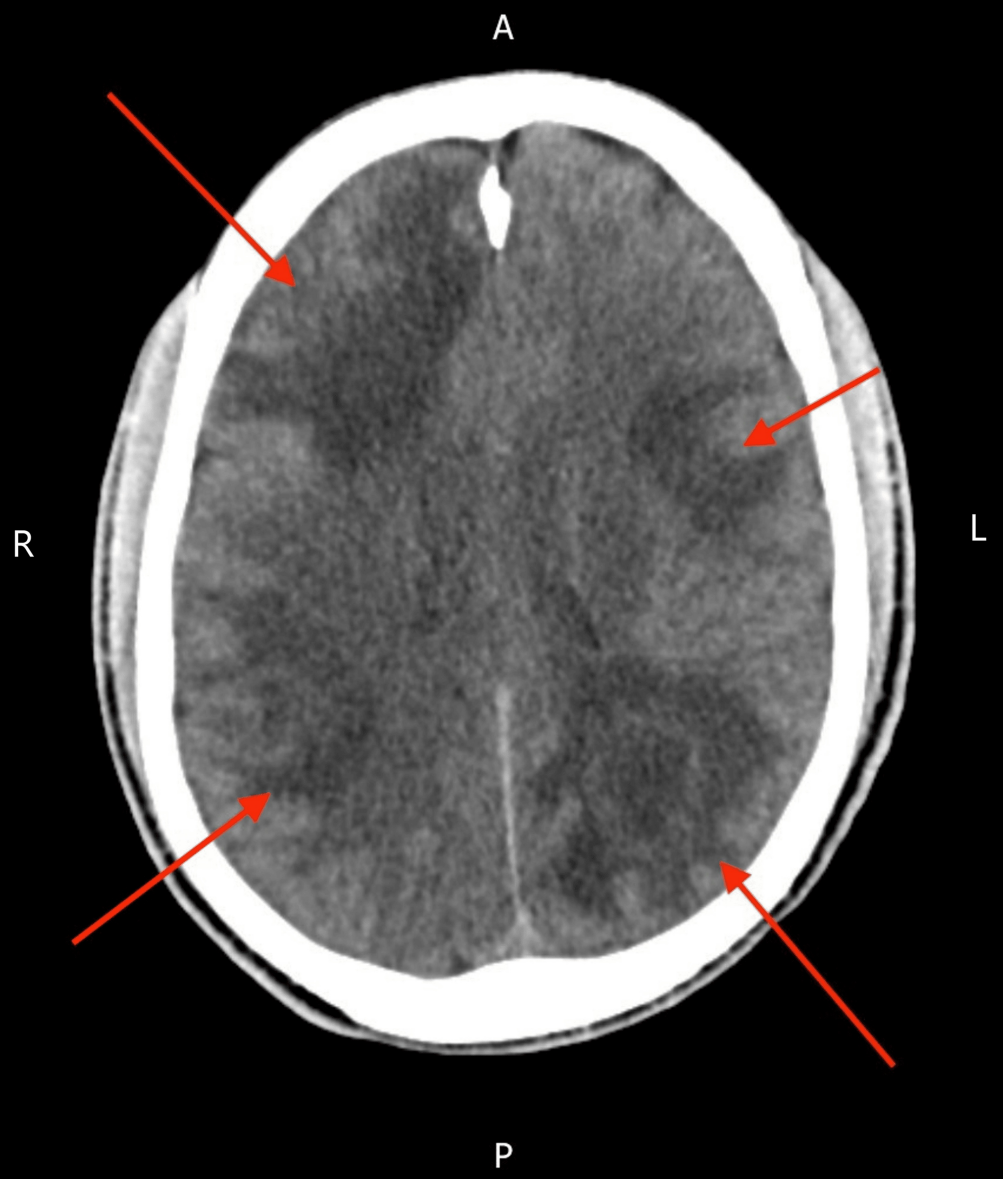
Non-contrast CT of the head showing multifocal oedema throughout both cerebral hemispheres with sulcal effacement CT: computed tomography

A non-contrast computed tomography (CT) scan of the head done on admission revealed multiple hypodense lesions with surrounding vasogenic oedema involving both cerebral hemispheres and the right cerebellum. There was sulcal effacement but no intracranial haemorrhage or infarction (Figure 1). These findings raised initial concern for intracranial metastases. A staging CT of the chest, abdomen, and pelvis showed no evidence of malignancy.

**Figure 2:**
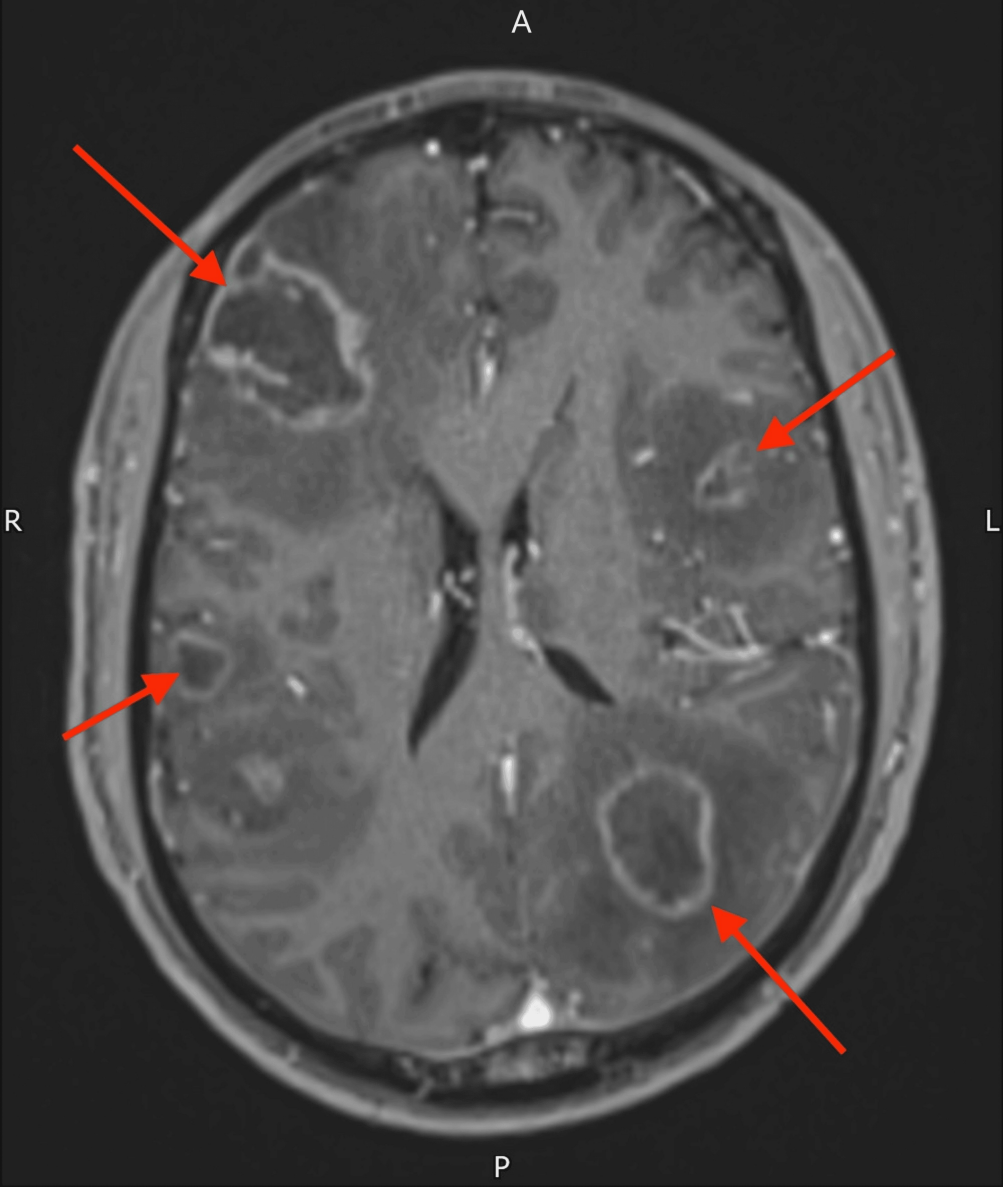
MRI of the head with contrast showing irregular ring-enhancing lesions in both cerebral hemispheres with surrounding vasogenic oedema MRI: magnetic resonance imaging

Subsequent contrast-enhanced magnetic resonance imaging (MRI) of the brain, two days later, demonstrated multiple ring-enhancing lesions in the basal ganglia and subcortical white matter, surrounded by extensive oedema (Figure 2). Given the absence of a known primary malignancy and the patient's age and demographic background, the radiologist recommended considering opportunistic infections, such as cerebral toxoplasmosis, in the differential diagnosis. 

Dexamethasone (8 mg twice daily) was initiated promptly to reduce cerebral oedema. The patient underwent further evaluation for the underlying causes of immunosuppression. HIV testing returned positive within a few days after admission, with a ribonucleic acid polymerase chain reaction (RNA PCR) viral load of 4,265,795 copies/mL and a CD4 count of 10 cells/µL, consistent with advanced immunodeficiency. Serological testing for *Toxoplasma gondii* showed positive immunoglobulin G (IgG) and negative immunoglobulin M (IgM), indicating the reactivation of latent infection. 

Empiric treatment for cerebral toxoplasmosis was initiated following results, with sulfadiazine (1 g four times daily), pyrimethamine (200 mg loading dose followed by 50 mg daily), and folinic acid (15 mg daily). The patient initially remained disoriented during the first several days of therapy. 

**Figure 3:**
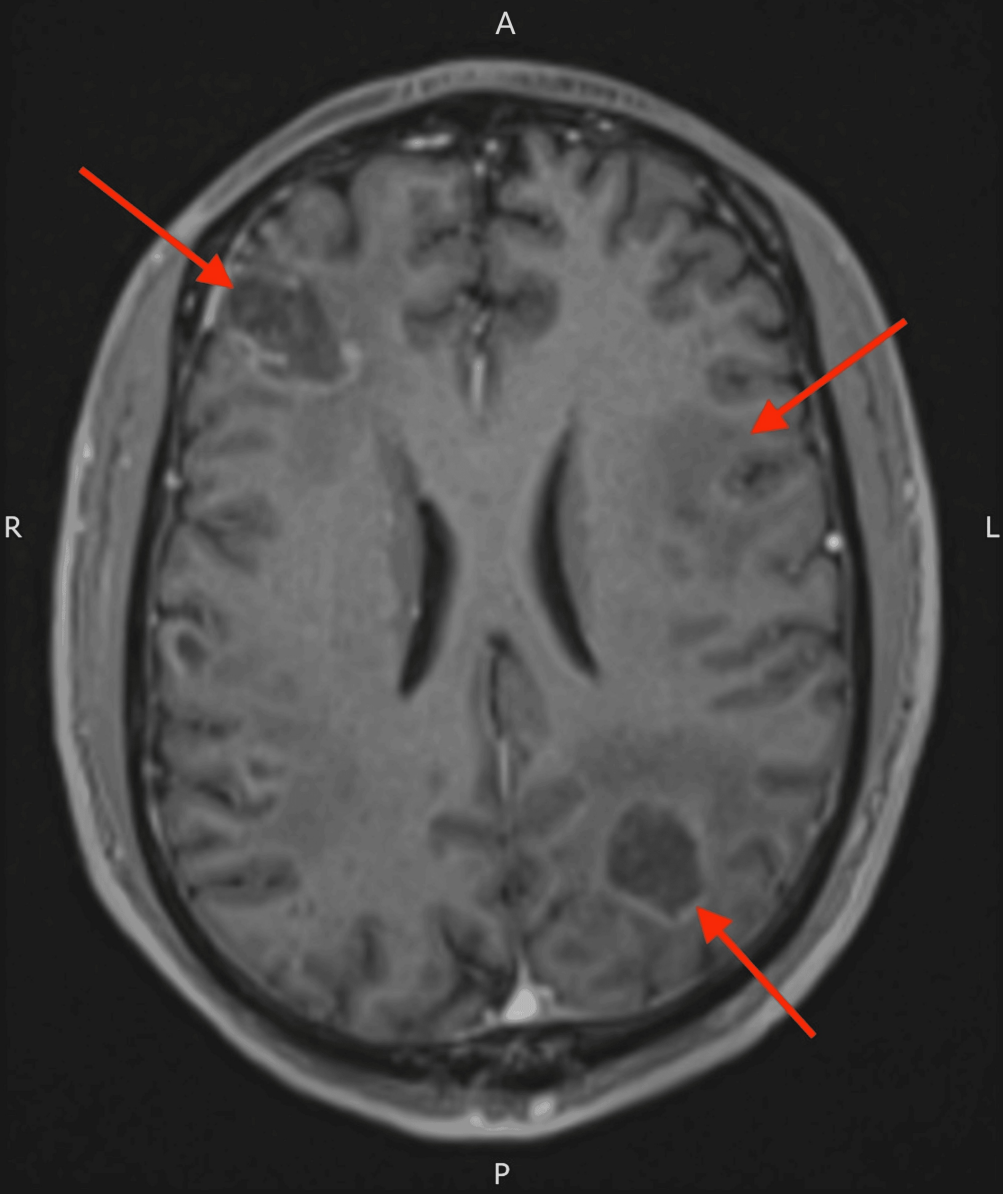
MRI of the head with contrast showing reduction in the size of the brain lesions and reduction in surrounding oedema MRI: magnetic resonance imaging

Two weeks after the initiation of treatment, follow-up MRI demonstrated a reduction in both lesion size and surrounding oedema (Figure 3), suggesting a favourable radiologic response. The patient's cognitive status improved in parallel, with recovery of verbal output, orientation, and attention. Once the patient was clinically stable, dexamethasone was gradually tapered, and antiretroviral therapy (ART) (Triumeq: 600-50-300 mg once daily) was commenced. Maintenance therapy for cerebral toxoplasmosis was planned following the completion of induction treatment. 

Additional viral and infectious screening revealed past cytomegalovirus (CMV) infection, resolved hepatitis B, and negative results for hepatitis C, syphilis, and cryptococcal antigen. 

The patient was discharged home in a stable condition with satisfactory bloods (Table 1), with arrangements for outpatient follow-up and continuation of secondary prophylaxis. 

## Discussion

This case illustrates a classic radiologic challenge: differentiating neoplastic from infectious brain lesions. In immunocompetent individuals, multiple ring-enhancing lesions most often indicate metastatic disease [1]. However, in patients with advanced HIV/AIDS, the diagnostic focus must shift. The most common etiologies for ring-enhancing lesions in such patients are *Toxoplasma gondii* encephalitis and primary CNS lymphoma [2]. Notably, patients from sub-Saharan African regions are at an increased risk of HIV and *Toxoplasma* co-infection [6]. Our patient, also from this region, who did not report any unprotected sexual activity since arriving in the UK, falls within this risk bracket. 

MRI in CNS lymphoma typically reveals a solitary lesion. In contrast, cerebral toxoplasmosis usually presents with multiple lesions, most commonly located in the basal ganglia, thalami, and corticomedullary junctions [7]. These lesions characteristically show ring or nodular enhancement with extensive surrounding vasogenic oedema. The distribution and radiological pattern observed in our patient were consistent with this, reinforcing the clinical decision to prioritise infectious over malignant etiologies. 

A well-known radiologic hallmark of cerebral toxoplasmosis is the "eccentric target sign", an asymmetrical nodular enhancement along the inner margin of the ring-enhancing lesion, which is considered highly specific for the condition [8]. A more recently described and potentially more specific marker is the "concentric target sign" on T2-weighted MRI, characterised by alternating concentric layers of hypo- and hyperintensity. This sign, typically found in deep parenchymal lesions, is considered more reliable than the eccentric target sign in differentiating cerebral toxoplasmosis from other brain lesions in immunocompromised patients [8,9]. 

Diffusion-weighted imaging (DWI) and apparent diffusion coefficient (ADC) mapping may provide further diagnostic value. Toxoplasmosis lesions often demonstrate increased ADC values, reflective of necrotic cores, whereas CNS lymphoma shows restricted diffusion due to its high cellularity [10]. Functional imaging, such as fluorodeoxyglucose-positron emission tomography (FDG-PET), can also aid differentiation; lymphoma lesions are typically hypermetabolic, while toxoplasmosis lesions tend to be hypometabolic [11]. Although these modalities were not employed in this case, they remain useful adjuncts in complex presentations. 

The diagnostic turning point in this case was the radiologist's consideration of an opportunistic infection in the absence of a definitive primary malignancy. Although the imaging findings alone could not definitively distinguish between malignancy and infection, the radiologist's differential diagnosis prompted further investigations, thus revealing advanced HIV and a positive *Toxoplasma* IgG. Given the clinical context, initiating a therapeutic trial was appropriate, as empiric treatment is often recommended before invasive procedures such as a brain biopsy, which is typically reserved for cases unresponsive to initial therapy [7]. Although in some unresponsive cases, it may seem as though empiric treatment was needless, as mentioned, it may avoid more aggressive investigations in most cases. The patient's clinical and radiologic improvement confirmed the diagnosis and obviated the need for biopsy.

Toxoplasmosis is significantly more prevalent among HIV-positive individuals with CD4 counts below 200 cells/mm³ than those with higher counts [12,13]. Ongoing maintenance therapy is necessary until immune reconstitution is achieved with ART, as the risk of relapse remains high in immunocompromised individuals [4]. Given our patient's severely depressed CD4 count of 10 cells/μL, he received a six-week induction course followed by maintenance therapy. ART was initiated once radiologic improvement was evident on follow-up MRI. 

In summary, this case underscores the critical role of radiologic interpretation in guiding timely and potentially life-saving interventions. Recognising the imaging hallmarks of cerebral toxoplasmosis, even in patients without a known immunocompromised status, can significantly alter the clinical trajectory and improve outcomes [14]. 

## Conclusions

This case highlights the pivotal role of radiology in shaping diagnostic pathways and enabling timely, vital interventions. In patients presenting with multiple ring-enhancing brain lesions, particularly those without a known HIV diagnosis, cerebral toxoplasmosis must remain a key differential. Radiologists play a critical role in identifying imaging features that help distinguish infectious processes from malignancy, thereby preventing unnecessary invasive procedures. In this instance, early radiologic insight prompted the consideration of an opportunistic infection, leading to a targeted therapeutic approach with favourable clinical and radiologic outcomes. Follow-up imaging not only confirmed treatment response but also guided the safe initiation of antiretroviral therapy. This case underscores the importance of radiologic awareness in immunocompromised populations and reinforces the value of neuroimaging in both diagnosis and longitudinal care planning. 
